# Analysis of Heavy Metal Sources in the Soil of Riverbanks Across an Urbanization Gradient

**DOI:** 10.3390/ijerph15102175

**Published:** 2018-10-04

**Authors:** Shudi Zuo, Shaoqing Dai, Yaying Li, Jianfeng Tang, Yin Ren

**Affiliations:** 1 Key Laboratory of Urban Environment and Health, Institute of Urban Environment, Chinese Academy of Sciences, Jimei Avenue 1799, Xiamen 361021, China; sqdai@iue.ac.cn; 2 University of Chinese Academy of Sciences, Beijing 100049, China; 3 Ningbo Urban Environment Observation and Research Station-NUEORS, Chinese Academy of Sciences, Ningbo 315800, China; yyli@iue.ac.cn (Y.L.); jftang@iue.ac.cn (J.T.)

**Keywords:** soil heavy metals, quantitative source apportionment, principal component analysis-multiple linear regression (PCA-MLR), GeoDetector model, GIS spatial analysis method

## Abstract

Regional soil quality issues arising from rapid urbanization have received extensive attention. The riverbank that runs through a city is representative of urbanization gradient transformation. Thirty soil samples in the Yangtze River Delta urban agglomeration were collected and analyzed for the concentrations of seven analytes. Correlation, principle component analysis, cluster analysis and GeoDetector models suggested that the four groups (Cr-Ni-Cu, Cu-Zn-As-Sb, Cd and Pb) shared the same sources in the core urban region; five groups (Cr-Ni-Cu-Zn, As, Cd, Sb and Pb) in the suburbs and three groups (Cr-Ni, Cu-Zn-Cd-Sb-Pb and As) in the exurbs. GeoDetector methods not only validated the results of the three other methods, but also provided more possible impact factors. Besides the direct influences, the interaction effects among factors were quantified. Interactive combination with strong nonlinear increment changed from between-two-weak factors in the central region to between-strong-and-weak factors in the suburbs. In the exurbs, the stronger interaction effects were observed between strong and weak factors. Therefore, the GeoDetector model, which provided more detailed information of artificial sources could be used as a tool for identifying the potential factors of toxic elements and offering scientific basis for the development of subsequent pollution reduction strategies.

## 1. Introduction

City managers pay extensive attention to the quantity and quality of the scarce resource in city land. Different types of land use and functional divisions of land in cities can lead to changes in the soil environment and even to soil pollution, which has also drawn increasing attention [1]. For example, farmland soil pollution may lead to food pollution, ultimately affecting human health. A well-known case is the Cd pollution of rice. Apart from renal dysfunction, cadmium poisoning is also found to result in carcinogenic, mutagenic and teratogenic effects. Long-term ingestion can cause “bone pain”, anemia, hypertension and emphysema. Lead poisoning has a negative impact on children’s intellectual development, as well as neuropathy, blood, hematopoiesis, digestion, cardiovascular and urinary system. Excessive intake of Cr, Zn and Cd (although these are some essential elements for the human body) can lead to diseases, such as digestive disorders (caused by chromium), respiratory diseases, abdominal pain, vomiting, anorexia and burnout (caused by zinc) and hemolysis, liver and gallbladder damage (caused by copper). Soil pollution will cause damage to other living organisms, ground water and sediments [2]. Due to the scale of urban development, history, population, leading industry and functional partition, different areas of the city carry out various functions. At the same time, soil pollution of the core urban, suburban and exurban areas becomes both a source and a sink of potential toxic elements and the ingredients influence one other [3]. Therefore, the soil pollution of different urban gradients has a certain commonality. Research on soil pollution from potentially toxic elements was conducted over the past few decades due to the development of multivariate statistical and GIS-based approaches for source identification in soils [3]. These elements mainly come from natural weathering of parent rocks, pedogenesis and human activities (e.g., urban-industrial expansion, fossil fuel combustion and agricultural practices). Many studies have confirmed that human factors are the main factors affecting soil pollution [4]. Under different local environmental conditions, human activities have different influences on the intensity and mechanism of action of soil pollution. As the intensity of human activity changes, soil pollution in different urban gradient regions is bound to be different too. Since urban environments are the most intense areas of human activity, studies on the current condition, sources of soil pollution and the main influencing factors of soil pollution under different urbanization gradients could provide a theoretical foundation and data for the formulation of subsequent mitigation management strategies.

The spatial distribution of potential toxic elements in soil is usually generated by interpolation of sampling point data and geostatistical methods. The combination of multivariate statistics and geostatistical analysis is favorable for identifying the impact factors of the spatial patent of heavy metals and distinguishing the natural and anthropogenic sources [5]. Scholars often use correlation analysis, principal component analysis (PCA) and its transformation forms (e.g., comprehensive constrained multivariate analysis methods (MULTISPATI-PCA)), and cluster analysis (CL) combined with different interpolation methods (spatial autocorrelation, kriging interpolation and its transformation forms and inverse distance weighting). The abovementioned three methods are applied to the source apportionment of the heavy metals, the spatial distribution uncertainty assessment, risk assessment and so on [6,7,8,9]. However, combinations of geostatistical analysis and multi-source statistical analysis are loose [5]. For example, Chen et al. improved the kriging interpolation method based on soil spectral information to obtain more accurate spatial distribution maps, and then used multi-statistical methods to analyze the sources [7]. Lu et al. further analyzed the sources of pollution after spatially interpolating the principal components obtained by the PCA method [9]. Hou et al. published a review article indicating that this loose integration method could also perform heavy metal source analysis, but was not able to accurately target specific human activity sources. Hou et al. also pointed out that besides the improvement of the interpolation precision of spatial distribution map by geostatistical methods, new methods should be developed to favor the combination of multivariate statistical analysis and geostatistical analysis in order to provide more precise guidance for improving soil remediation strategies in the future [5]. The GeoDetector model used in this study was a method based on spatial stratification (heterogeneity) from geostatistics theory, which takes the spatial similarity between the influencing factors (independent variables) and the spatial distribution of a heavy metal (dependent variable) into consideration. The model delineates the interaction between each variable, quantitatively analyzes the type of variables, and forms a more quantitative and accurate framework than the source apportionment method with multivariate statistical analysis. The model provides an effective method for clarifying the mechanism of spatial distribution of heavy metals in urban soils [10,11].

China’s Yangtze River Delta urban agglomeration is a region with some of the fastest urbanization and economic growth in the world. Soil pollution problems in this area are gradually emerging [12,13]. Soil pollution has raised concerns about the quality of agricultural products [2,14]. According to a survey by China’s environmental protection department, in 2011, China’s arable land contaminated by the potential toxic elements such as cadmium, arsenic, chromium and lead amounted to nearly 150 million mu, accounting for about one-tenth of the total cultivated land area [15]. Another threat comes from the industrial pollution emissions brought about by economic development [16]. China’s 2014 soil pollution status bulletin showed that soil pollution in the south was heavier than in the north. The Yangtze River Delta and the Pearl River Delta were more seriously affected [17]. As the Yangtze River Delta urban agglomeration is under a multi-centered development mode, urbanization gradients intensively and repeatedly alternate between “high”, “medium”, and “low”. The soil quality changes from the urban core to the exurbs, which has attracted the attention of city managers. The environmental security of the suburbs is particularly important because they supply food, vegetables and fruits for the urban area. However, due to the large area occupied by the industrial and mining enterprises, farms, waste treatment stations and traffic arteries, the sources of potential toxic elements in soil are very complicated. Though some research has been conducted in order to determine the concentration and spatial distribution of heavy metals in the soils around cities [18], there are few studies on the characteristics and sources of heavy metals along urban gradients so far.

Therefore, we chose the relatively independent Zhangxi stream region of Ningbo City in China as the study area and collected surface soil samples along the changing urbanization gradient and described the spatial distribution of heavy metals. A total of 30 soil samples were collected and analyzed for Cr, Ni, Cu, Zn, As, Cd, Sb, and Pb. Combined correlation analysis, PCA and CL analysis methods of multivariate statistical methods and a GeoDetector model were employed to investigate the current state of soil heavy metal contamination in the study area and to identify the possible sources and impact factors of the heavy metals. The multivariate statistical methods and the geostatistical methods proposed in this study complemented each other, leading to more precise guidance for the formulation of soil protection policies.

## 2. Materials and Methods

### 2.1. Study Area

**Figure 1:**
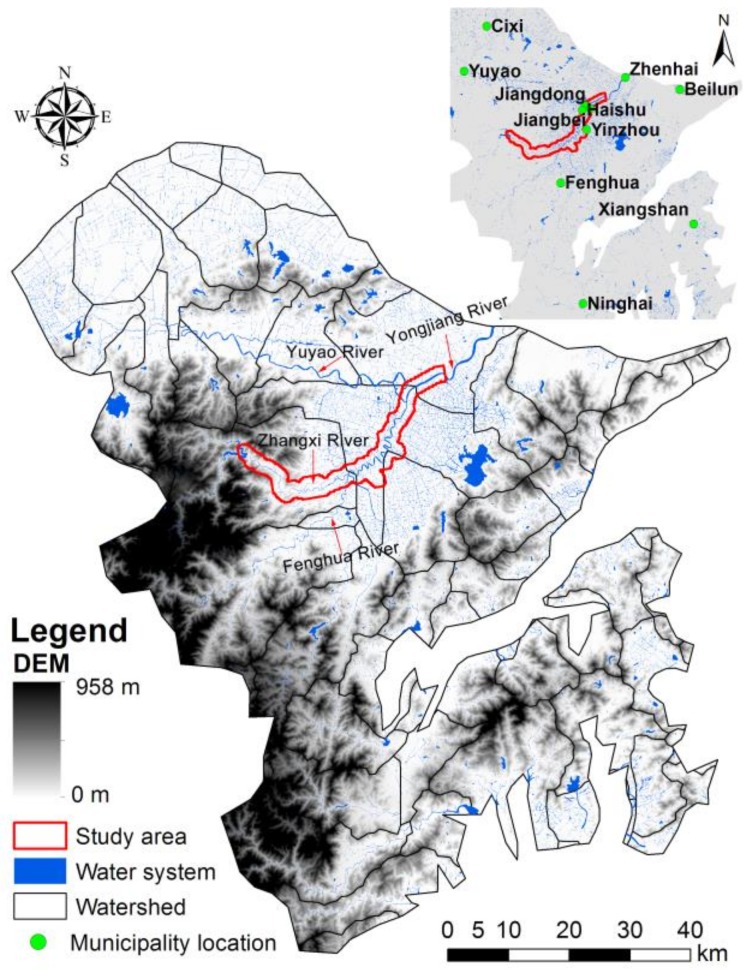
Locations of the study area, the water system, municipal governments of districts in Ningbo City.

Ningbo, the capital of Zhejiang Province, is located in the Yangtze River Delta urban agglomeration area on the southeast coast of China. The urban region includes the Zhenhai, Beilun, Jiangbei, Yinzhou and Haishu Districts. The terrain of Ningbo is high in the southwest and low in the northeast. It belongs to the subtropical monsoon climate area, which is mild and humid with four distinct seasons. The average annual temperature is 16.4 °C; the average annual precipitation is about 1480 mm, and the average annual sunshine hours are 1850 h. Ningbo has one of the eight major water systems in Zhejiang Province, where the rivers include the Yuyao River, Fenghua River and Yongjiang River. Yuyao River originates from Lianghu Lake in the Shangyu District of Shaoxing City in the northwest. Fenghua River originates from the Banzhu of Fenghua District in the south. The source of Fenghua River is far from Ningbo City, and it flows through many urban areas. The two rivers join together as the Yongjiang River in the “Sanjiangkou” of the urban area of Ningbo city, and flow northeast into the East China Sea. Zhangxi River originates in the hinterland of Siming Mountain in the west, which is close to Ningbo City. It flows through the exurbs, suburbs and urban core area of Ningbo city and is then diverted in Tuoshan Yan in Qijiang Town, which used to be a famous ancient water conservancy project. The one tributary of Zhangxi River is called the Yinjiang River and is injected east into the Fenghua River. The other tributary enters the urban area of Ningbo city along the Nantang River. The Zhangxi River was not affected by other cities in the Yangtze River Delta urban agglomeration, and could better represent the different stages of urbanization experienced in an urban system (Figure 1). In 2017, we divided the Zhangxi River into upstream (exurban), midstream (suburban) and downstream (the core area) zones according to the ratio of impervious area, and collected 30 surface soil samples along that line. There are huge differences between the urban and non-urban system, such as the intensity, structure and components. Moreover, the suburb region, as the transitional zone, faces a series of environmental problems because of the urban expansion and inappropriate planning. These issues have drawn increasing attention of scholars. The division of the study area into three regions helps to clarify the various human activity intensity and category that lead to the soil pollution problems.

There are three types of soil parent material in Ningbo: red loam developed by igneous rocks and slope sediments, rice soil developed by fluvisols, and salt soil and rice soil by marine sediments. Soil textures of the study region are clay, silty clay loam, silt loam, loam and loamy sand (Supplementary Materials Figures S1 and S3). In the core urban region of the study area, the land use type is construction land. The soil textures are clay, silty clay loam, silt loam, loam. The soil types are acrisol (soil with subsurface accumulation of low activity clays and low base saturation), anthrosols (soils in which human activities have resulted in profound modification of the soil’s properties). In the suburb, the farmland is the major land use; The soil texture includes clay, silt loam, loam; Soil type includes acrisol, anthrosols and alisols (soil with sub-surface accumulation of high activity clays, rich in exchangeable aluminum). The forest and rural residential locations occupy the exurban region. The soil texture includes silt loam, loam and loamy sand; Soil type includes acrisol, anthrosols, fluvisols (young soil in alluvial deposits) and regosol (soil with very limited soil development).

### 2.2. Sample Collection and Measurement

In order to obtain 30 representative farmland soil samples (depth: 0–20 cm), soil samples were collected and stored in accordance with the Technical Specifications for Soil Environmental Monitoring (HJ/T166-2004) in China. See Supplementary Materials Table S1 for the locations. Firstly, the natural landscape of the sampling point should meet the requirements of the soil environmental background value study. The sampling points were selected in places where the type of soil was particularly obvious, the terrain was relatively flat, stable, and with good vegetation. The sampling points were not located in places with slopes, depressions and other subordinate landscape features. The sampling points were located where a well-developed profile with a clear level and intrusive body was evident. The sampling points were not located where the soil erosion was serious or the topsoil was destroyed. The sampling points were not set up in the small marginal areas where various soils and parent mother rocks were interlaced. Secondly, the collection of mixed samples adopted the plum point method, which was applicable to plots with small area, flat terrain, relatively uniform soil composition and degree of contamination. Five points were set up in this area. We mixed samples from each point and then took 1 kg of soil sample into the sample bag by the quadruple method. Finally, the soil that contacted the metal sampler was sliced off using bamboo strips or knives.

We recorded the geographical coordinates of the sampling points, including altitude, slope, land-use type, etc. and took photos. The samples collected were brought back to the laboratory for air-drying on the day of sampling. Some samples were passed through a 20-mesh sieve for pH and cation exchange capacity (CEC) determination. Others were passed through a 100-mesh sieve for the determination of organic matter and heavy metals. Then, 0.2 g of the sample was accurately weighed (accurate to 0.0002 g) using a BSA124s balance (Sartorius, Goettingen, Germany) and placed in a digestion tube, to which 6 mL of nitric acid (high purity grade), 2 mL of hydrochloric acid, and 1 mL of hydrofluoric acid were added. The tube was then placed on an electric heating plate in a fume hood and preheated to 80 °C for 20 min. Next, the tube was taken out and placed in a microwave digestion apparatus (CEM MARS 6 Classic, Matthews, NC, USA) for digestion. After the digestion process, cooling and evaporation were carried out at a temperature of 140 °C for about 3 h, until tube bottom could be seen with the naked eyes. We then slightly cooled the tube, transferred the digestion solution to a 50 mL centrifuge tube, and diluted the sample to 30 mL with ultrapure water. The sample was well shaken and measured by ICP-MS. ICP-MS instrument (ThermoFisher ICAP Q, Waltham, MA, USA) Parameter settings were as follows: atomizer: high salt atomizer; sampling depth: 6 mm; transmitting power: 1300 w; carrier gas flow 1.15 L/min. As to other parameters the analysis by ICP-MS, please see Supplementary Materials Table S2. In the process of determination, standard soil GBW07454 (provided by the Institute of Geophysical and Geochemical Exploration, Beijing, China) and three parallel samples were used for the quality control. If the standard soil sample in a batch of samples was not qualified, the batch of samples would be measured again.

### 2.3. Statistical Data Analysis

The concentration values of seven analytes in different urban gradients followed a normal distribution. Pearson correlation analysis accurately measures the linear relationship between two variables. The principal component analysis and cluster analysis that behaved similarly to identify potential sources were adopted to analyze the 30 samples data across the three urban gradients. Statistical analysis was conducted using SPSS 16.0 (SPSS Inc., Chicago, IL, USA). One-way analysis of variance (ANOVA) was used to compare data from different gradients of soil samples. The potential pollution source conclusion was not only based on the Pearson, PCA, and CL results, but also consulted the GeoDetector results which were analyzed on the 3 434 fishnet polygon data (30 × 30 m resolution).

In regard to interpolation of the spatial distribution of analytes’ concentrations, Hou et al. reviewed the analysis methods of heavy metal soil pollution at regional scales. They pointed out that the methods used to interpolate the spatial distribution map of soil heavy metals included kriging as well as its variant methods, and inverse distance weighting (IDW) interpolation methods [5]. Liao et al. compared and analyzed six spatial different methods including kriging and its variant methods, IDW, etc., and then summarized that IDW was appropriate for the conditions of large spatial scale, high spatial autocorrelation and low sampling proportion [19]. On the basis of analyzing the distributions of sampling points in soil heavy metal interpolation in published articles, the statistical results of Hou et al. revealed that the density of sampling points per square kilometer was between 0.0004 and 6.1, with an average of 0.4 [5]. They argued that low density sampling could be used in areas where the distribution space varied little, and that higher densities should be adopted in areas with much larger variation in terms of distribution space. For instance, 3.2 and 3.7 samples per km^2^ were used by Li et al. [20] and Lee et al. [21], respectively. In terms of this conclusion, we used a 1 km buffer range along the banks of the Zhangxi River as an interpolated study area. Cross validation Arcmap software (ESRI, RedLands, CA, USA) was used to verify the accuracy of the map.

### 2.4. Multiple Source Data Integration and Geographical Detector Method

After the availability of the spatial distributions of eight heavy metals, a geographical polygon database was created in order to integrate multi-sources impact factors data. Finally, the GeoDetector model could be applied to interfere the individual and interactive influences of the selected impact factors on the distributions of the heavy metals’ concentrations.

#### 2.4.1. Multiple Source Data Integration

We analyzed the main factors influencing the heavy metals, based on the existing studies [6,22,23,24,25]. There were four categories of factors from five different sources. The main factors included the topography (elevation, slope position, slope direction and slope degree from DEM data), soil characteristic (pH, NH_4_^+^, NO_3_^−^-N, organic matter, soil texture, humus depth, and soil site index) and green land characteristics (area, dominant species, and stand age), anthropogenic activities (first class and second class land use type, agriculture area, industry area, transportation area, rural living area, and nighttime light intensity) The 30 m digital elevation model (DEM), the first data source, was downloaded from Geospatial Data Cloud (http://www.gscloud.cn/). This data source provided the topography indicators. The soil and green land characteristics were from two other data sources. They were the spatial interpolated maps based on 30 collected samples and 2016 Forest Management Planning Inventory (FMPI) obtained from Ningbo Forestry Bureau, in which forest characteristics (patch area, stand age, and dominant tree species) and soil characteristics (soil depth, humus depth, soil texture and site index) were found. The soil textures are divided into sand, loam and clay. The site index (high, median and low class) refers to the rank of forest productivity based on the relationship between the average height of stand and the average age of stand. The FMPI data are collected every 10 years by the forestry administration in China and widely used in the forest ecology monitoring and research [26]. The fourth data source was the land use vector map in 2016 which was comprised of six first-class types (agricultural land, grassland, forest, water body, urban and unused land) and 17 second-class types, which divide the first-class delineations into more detailed functional zones. The data were commercial product retrieved from Landsat 8 images and validated by the visual interpretation. The fifth data source was 2016 NPP-Visible Infrared Imaging Radiometer data (NPP-VIIRS) of the study area downloaded from National Oceanic and Atmospheric Administration (NOAA, https://ngdc.noaa.gov). The simple correction method for nighttime light adjustment in NPP-VIIRS data unified the negative value into 0 and resampled it to 500-m resolution to correct the data. Population density distribution could be inferred accurately by the night time light data, which represented the human activity intensity [27]. The core for the interpolation was the weights of population density from digital number (DN) value obtained through the remote sensing satellite.

The impact factors data included the vector and raster data which differed in structures and forms. To create a multi-source dataset with uniform coordinate, we used the correcting methods (e.g., format conversion, coordinate transformation, and geometric correction) on different types of data. The 3 343 30 m × 30 m fishnet was used as a basic unit of analysis. Then, the zonal statistics function of Arcmap 10.1 was used to calculate the average values for each pixel. Finally, the fishnet data intersected with the FMPI of vector data. After this step, we got the final input data of GeoDetector. Each polygon of this data had the same attribute, including four categories of factors: topography, soil, green space and human activity.

#### 2.4.2. Geographical Detector Method

The geographical objects are always characterized by spatial stratified heterogeneity or autocorrelation. When the sum of the variance of sub-areas is less than the total variance of the whole study area, the stratified heterogeneity exists. The statistical correlation could be detected through the similarity of the spatial distributions of the two variables. GeoDetector is a statistical method with no linear hypothesis to detect spatial stratified heterogeneity and reveal the similarity of factors. According to three urbanization gradients, we used the factor detector module of the GeoDetector method to analyze the influence of a single dominant driving factor that caused the spatial distribution of heavy metals, and determined the significance level using the *p* value of the F test [28]. The high influence force q value (Equation (1)) illustrated the high degree of similarity and a strong spatial correlation between the influence factors and the heavy metal concentrations (http://www.geodetector.org/):

(1) $$q_{x}=1-\frac{\sum_{p=1}^{m}n\sigma_{D,p}^{2}}{N\sigma_{D,z}^{2}}$$

In the above equation, q*_X_* represents the determinant power of each impact factor; hypothesis D is a potential impact factor; n is the number of samples in the sub-region affecting factor D; N is the number of samples in the entire region; m is the number of impact factors; $\sigma_{D,p}^{2}$ is the variance of the variable in the D sub-region; and $\sigma_{D,z}^{2}$ represents the discrete variance across the study area. Assuming $\sigma_{D,z}^{2}\neq 0$, the model is established. The interval of q*_X_* value is [0, 1], and the q*_X_* value is $1-\frac{\mathrm{sum}\;\mathrm{of}\;\mathrm{discrete}\;\mathrm{variances}}{\mathrm{discrete}\;\mathrm{variance}\;\mathrm{of}\;\mathrm{population}}$. The large value indicates that the explanatory variable factor has a great influence on the explained variable.

When two or more factors act together on an event, they are affected by each other. Besides that, the displayed effect on the event is significantly different from the sum (and/or) product of two or more factors acting alone, which is called an interaction effect between these factors [29]. In traditional statistical analysis, significant interaction effects distorted the main effect results. Therefore, it is necessary to use simple effect analysis to test whether the influence of significant single factors is exaggerated by significant interactive effects [30]. The interaction effect of two factors measured by interaction module of GeoDetector is to compare the sum of q values of the X_1_ and X_2_ factor with that of X_1_ ∩ X_2_. The comparison results include linear or nonlinear mutual enhancement, attenuation and independence. The principle of the interactive detector module is:

(2) $$\begin{array}{c} \mathrm{Weaken},\mathrm{nolinear}—:\;q\left(X_{1}\cap^{}X_{2}\right)<\mathrm{Min}\left(q\left(X_{1}\right),q\left(X_{2}\right)\right)\\ Weaken,uni—:\;\mathrm{Min}\left(q\left(X_{1}\right),q\left(X_{2}\right)\right)<q\left(X_{1}\cap^{}X_{2}\right)<\mathrm{Max}\left(q\left(X_{1}\right),q\left(X_{2}\right)\right)\\ Enhance,bi—:\;q\left(X_{1}\cap^{}X_{2}\right)>\mathrm{Max}\left(q\left(X_{1}\right),q\left(X_{2}\right)\right)\\ Indepent—:\;q\left(X_{1}\cap^{}X_{2}\right)=q\left(X_{1}\right)+q\left(X_{2}\right)\\ Enhance,nonlinear—:\;q\left(X_{1}\cap^{}X_{2}\right)>q\left(X_{1}\right)+q\left(X_{2}\right) \end{array}\}$$

X_1_ and X_2_ are two selected independent variables. X_1_ ∩ X_2_ refers to the new factor produced by overlapping the X_1_ factor and the X_2_ factor, i.e., the interaction effect of the two factors. The concentrations are the dependent variables. The interaction detector module of GeoDetector software compares the q values with the new factor and the existing independent variable, which was different from the traditional statistical analysis hypothesis and test method. The result could be used as the criteria for the retention of effect-modified factors, which had a small single impact but produced the dominant interaction influence. When a single factor had a large direct and interactive effects with other factors at the same time, it should be the dominant factor. The GeoDetector method is particularly good at analyzing category variables. As to the continuous variable, we used the Jenks natural breaks classified method in order to differentiate the stratification of factors.

## 3. Results

### 3.1. Descriptive Statistics of Heavy Metal Concentrations in the Different Urban Gradients and Background Values

**Figure 2:**
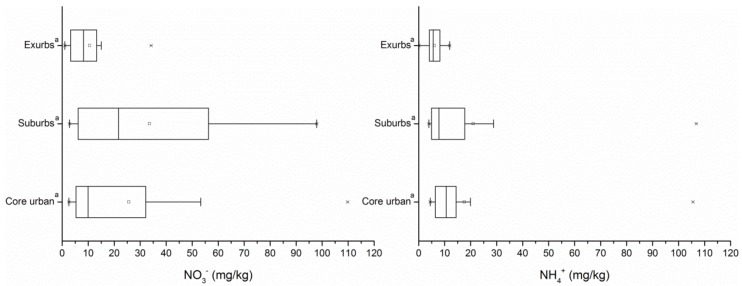
The changing trends of NO_3_^−^-N and NH_4_^+^ across urbanization gradients. The same letter “a” indicates no significant differences across the urban gradients at a *p* < 0.05.

The maximum pH value of the study area was 8.40; the minimum value was 4.77, and the average value was 7.42 ± 0.85. The pH distribution map showed an area of weak acidity in the outer suburbs and an area of weak alkalinity in the suburban and central urban areas. The contents of organic matter were high in the exurbs (mean ± SD = 52.78 ± 27.89), low in the suburbs (32.26 ± 14.95) and central areas (35.48 ± 18.71). The nitrate content was the highest in the suburbs with large variability. See Figure S2 in the Supplementary Material for the spatial distributions of general characteristics of soils. The average content of ammonium ions did not differ significantly along different city gradients (Figure 2).

**Figure 3:**
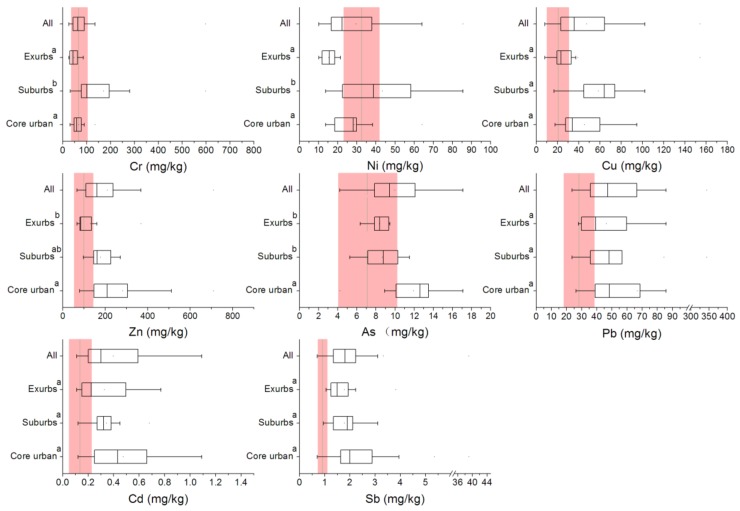
The background values (arithmetic mean ± stand deviation, red region) and trends in terms of changes in the concentrations of eight heavy metals across urbanization gradients. The same letter “a and b” indicates no significant differences across the urban gradients at *p* < 0.05.

Figure 3 displays the descriptive statistics of Cr, Ni, Cu, Zn, As, Cd, Sb and Pb concentrations in the topsoil at the 30 sampling locations among different urban gradients. Clearly, the concentrations of different heavy metals showed considerable variation across the study area. Concentrations of seven heavy metals (Cr 92.92 ± 109.02 mg/kg (mean ±SD), Ni 29.51 ± 19.15 mg/kg, Cu 47.72 ± 32.51 mg/kg, Zn 210.5 7 ± 151.04 mg/kg, As 10.00 ± 3.05 mg/kg, Cd 0.40 ± 0.26 mg/kg, Sb 3.33 ± 6.90 mg/kg, and Pb 66.67 ± 64.63 mg/kg) grew significantly compared with the background values obtained from the “The soil element background values in China” report in 1990, except for Ni, in all regions [31]. The samples of the background values were uniformly distributed in the cities, which were for A layer soil (0–20 cm). See Table S3 in the Supplementary Material for detailed information of background values in Ningbo City. In terms of the various urban gradients, Cr, Ni and Pb concentrations in exurban regions were lower than the background mean values. The concentration of Ni in the core urban region was even lower. Cr, Ni, As and Pb were lower than the class II levels of the soil environment quality standard (GB 15168-1995) [32] (Table 1). Cu in suburbs, Zn in core region, and Cd in three gradients were on the class II level. While there wasn’t value recorded in the soil environment standard (GB 15168-1995), Sb had significant increases compared with levels recorded in 1990. The core urban region had higher values for all the heavy metals except Cr and Ni. The suburb region had higher values for all the heavy metals except As. The exurb region had higher values for Pb, Cd and Sb. Therefore, we assumed that Cr, Ni and As were contributed by the natural sources.

### 3.2. The Spatial Distribution of the Heavy Metals during the Urbanization Gradients

**Figure 4:**
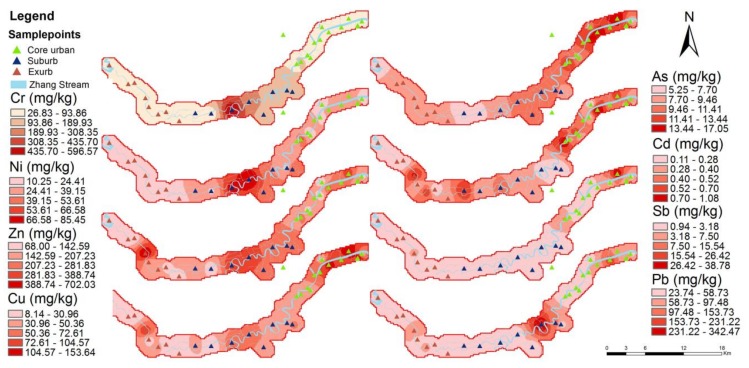
The spatial distribution of the heavy metal concentrations during the urbanization gradients.

The inverse distance weighted (IDW) interpolated spatial patterns of Cd, Ni, Cu, Zn, As, Cd, Sb and Pb concentrations in the topsoil of the study area are presented in Figure 4. 

Table 2 reports the accurate values. If the prediction errors are unbiased, the mean prediction error should be near zero. Figure 4 shows that the high Cr and Ni value areas were located in the suburban arable area closer to the main road s34. The high Cu and Zn values were distributed in three different urban gradients, but most of the values in the exurbs were low. The high concentrations of Cu and Zn in the core area were close to the main roads, automobile industrial areas, and soil polluted by factories. In the suburbs, these concentrations were close to the industrial park, and in the exurbs, they were close to the tea farm. The high values of As and Sb were mainly distributed in the central area and were close to the factory areas (automobile industry area). The high value of Pb in the suburbs appeared near Huang’ai Village in Haishu District, adjacent to the Ningbo Rail Transit Group Co., Ltd. headquarters, and was also surrounded by dyeing and finishing and machinery factories. The high Cd value in the central area was mainly distributed near roads and viaduct bridges, close to special steel mills, and factory-polluted land. The high-value areas in the exurbs were located in tea factories, cultivated areas and farmland areas near highways. See Figure S3 in the Supplementary Material for the land use, road network and factory locations information.

### 3.3. Source Apportionment for Heavy Metals

#### 3.3.1. Correlation Analysis

Table 3 is a summary of Pearson’s correlation coefficients between heavy metals and soil properties. In the core urban region, it can be seen that Cr had a significant positive correlation with Ni (r = 0.967 **) and Cu (r = 0.697 **). However, the element NH_4_^+^ exhibited positive correlations with Cr and Ni (r = 0.768 ** and 0.835 **, respectively), but non-significant correlations with Cu. This suggested that Cr, Ni and Cu might have different sources. Moreover, the element Cu was positively correlated with Zn, As and Sb (r = 0.695 **, 0.645 *, and 0.637 *), whereas they showed a positive correlation with OM. The elements Zn and As were correlated with each other (r = 0.680 *). In the suburban region, Cr showed a positive correlation with Ni, Cu and Zn (0.746 *, 0.735 *, and 0.731 * respectively). There was not any correlation between the rest of the four elements with any other elements. The element NH_4_^+^ was weakly correlated with Cd. In regards to the exurbs, Cr had a significant positive correlation with Ni (r = 0.956 **) as well. The element Cu was correlated with Zn, Cd, Sb and Pb (r = 0.988 **, 0.809 *, 0.970 **, and 0.855 **, respectively). There was a strong correlation among Cu-Zn-Cd-Sb-Pb group. The elements As and Pb didn’t correlate with other elements. The elements Ni and Sb showed a positive correlation with pH, and Cu. The element As showed a negative correlation with NH_4_^+^. Overall, the eight elements could be divided into different groups in different areas. In the core urban region, they should be divided into four groups: Cr-Ni-Cu, Cu-Zn-As-Sb, Cd, and Pb; In the suburbs, they should be divided into five groups: Cr-Ni-Cu-Zn, As, Cd, Sb, and Pb; In the exurbs, they should be divided into three groups: Cr-Ni, Cu-Zn-Cd-Sb-Pb and As.

In the whole study area, as we observed in the three urban gradients, the elements Cr, Ni, and Cu had significant correlations with each other (the range of r is 0.504 **–0.773 **). The element Cu also correlated with OM (r = 0.372 *) and Zn (r = 0.588 **). There was a similar significant correlations among Zn, As, and Sb (the range of r is 0.480 **–0.678 **). The element Cd correlated with both Zn (0.600 **) and Cu (0.444 **), but didn’t have a significant relationship with Pb and Sb. The element Pb was independent from others. Therefore, the group result from 30 samples could be defined as three groups: Cr-Ni-Cu, Zn-Cu-As-Cd-Sb, and Pb, which was basically consistent with the above separate analysis results. This method can’t provide further information to clarify the complicated relationship among Cu-Zn-As-Cd-Sb, which might be the reason of the different divisions comparing with the separate analysis results.

#### 3.3.2. PCA Analysis

Since the samples in suburbs and exurbs failed the Kaiser-Meyer-Olkin (KMO) measure and Bartlett’s test of sphericity factor analysis was conducted to identify the sources of heavy metals in core urban region and the whole 30 samples. Results showed that three factors were extracted, which could account for over 81.89% of the total variation of heavy metal concentrations.

The results listed in Table 4 indicate that, except for Pb, the remaining seven elements were strongly associated in the first factor (F1) with high loadings (larger than 0.5), explaining 50.02% of the total variance. Factor 2 (F2) was negatively dominated by Cr and Ni and positively controlled by Zn and Cd, which accounted for 19.31% of the total variance. The elements Zn and Cd were partially represented in F1 and F2 (loadings of 0.748 and 0.543 for Zn and 0.522 and 0.500 for Cd, respectively), suggesting complex influence sources. The third factor mainly condensed the information of Sb and Pb, which explained 12.56% of the total variance. Considering the relatively low levels of Cr, Ni, and As concentrations that had high scores in F1, we assumed that F1 may be the natural source. In terms of the samples in the whole study area, there were three factors as well. They shared similar variance proportions of explanation power compared with that in the core urban area, but the cumulative decreased because the deduction of F1. F2 was different from F2 in the core urban region. It was positively dominated by Cr and Ni, and negatively influenced by Zn and Cd with the decreased power. F1 and F3 had similar loadings with that in the core urban region. 

#### 3.3.3. Cluster Analysis

**Figure 5:**
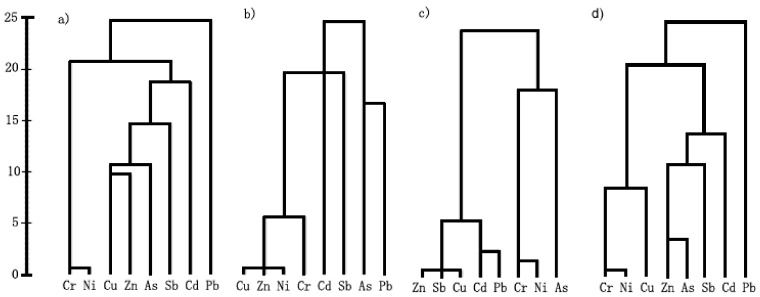
Dendrogram of the cluster analysis of soil heavy metals in (**a**) urban core region, (**b**) suburban region, (**c**) exurban region and (**d**) the whole region.

The dendrogram of CA (Figure 5) showed four clusters in the core urban region (Cr-Ni, Cu-Zn-As-Sb, Cd and Pb), four groups in the suburbs (Cu-Zn-Ni-Cr, Cd, Sb, and As-Pb), three groups in the exurbs (Cu-Zn-Cd-Sb-Pb, Cr-Ni and As), three groups in whole region (Cr-Ni-Cu, Zn-As-Sb-Cd, and Pb). 

The differences of CL results from correlation results were the classification of Cu in the core region and the combined group of As-Pb in the suburb. The whole region cluster result attributed the Cu with the Cr-Ni group.

#### 3.3.4. Geodetector Model

Table 5 shows the contributions of 17 factors to the soil concentrations of eight heavy metals in the core urban region. Among them, the soil characteristic was the dominant factor. Specifically, the influential factors of Cr and Ni were close in the core area. The main influential factors were soil (NH_4_^+^ was the largest), and human activity factor (second-class land use and nighttime light). The main influence factors on Cu of the Cu-Zn-As-Sb group were soil organic matter, pH, second-class land use and nighttime light. The dominant influencing factors of Zn were topography (elevation, slope direction and slope position), green land area and land use. The major influencing factors of As were soil (organic matter, soil texture, NH_4_^+^ and NO_3_^−^-N) and second-class land use. The influencing factors of Sb were soil factors (pH, organic matter, soil texture and humus depth), excluding topography. In regard to Cd, soil (NO_3_^−^-N, organic matter, soil texture, and humus depth) was a strong factor. Soil (organic matter and pH) was also a strong factor for Pb.

The influencing factors on the Cr-Ni-Cu-Zn group in the suburbs were consistently dominated by soil (NH_4_^+^, organic matter, NO_3_^−^-N and soil texture), topography and second-class land use. In the As-Pb group, NH_4_^+^, NO_3_^−^-N, soil texture, and nighttime light were great influencing factors for As. NH_4_^+^, NO_3_^−^-N, organic matter, nighttime light and agricultural land area were strong factors for Pb. In terms of Cd, the most influential factors were NH_4_^+^, NO_3_^−^-N, agricultural land area, and organic matter. For Sb, they were organic matter, NO_3_^−^-N, soil texture, nighttime light, and second-class land classification.

In the exurbs, the dominant factors of Cr and Ni were close. The dominant factors included soil (pH, NH_4_^+^, NO_3_^−^-N and organic matter), land use type, dominant tree species and agricultural land area. The element As was mainly affected by pH, NH_4_, organic matter, second-class land use and rural residential areas. The major factors for Cu, Zn and Sb including soil (pH, NH_4_^+^), rural residential areas and second-class land use were similar. The factors influencing Cd and Pb were the same, and similar to the Cu-Zn-Sb group. These factors were pH, rural residential area, second-class land use, and the dominant tree species.

In conclusion, in the central urban area, the major influencing factors on the Cr-Ni group not only included natural factors, but also included certain human activity factors. The various major factors of the Cu-Zn-As-Sb group indicated that the sources were similar but slightly different. In the suburbs, the major factors of the Cr-Ni-Cu-Zn group and the As-Pb group were relatively consistent, including both natural factors and human activity factors. For Cd, major factors included soil properties and farmland area, but for Sb, the contribution of human activity factors was also evident. The classification of similar sources in the exurbs was confirmed by Geodetector results, indicating that the influencing factors in the exurbs were relatively simple and easy to identify.

**Figure 6:**
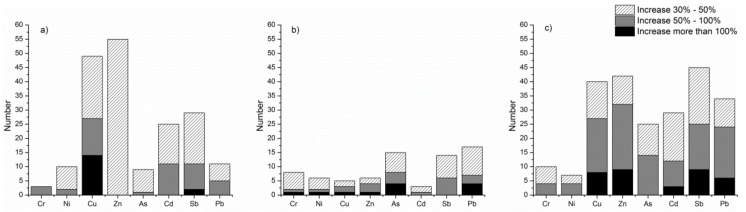
Numbers of nonlinear increase combinations in (**a**) urban core regions, (**b**) suburban regions, and (**c**) exurban regions. “Increase” means the comparison between the q value of interactions and the sum of two impact factors. “Increase 30–50%” low-level non-linear enhancement, “Increase 50–100%” means mid-level non-linear enhancement, “Increase >100%” means high-level non-linear enhancement.

From the results of the interaction between two factors given by the GeoDetector software (Figure 6), the combinations of interactive factors with nonlinear enhancements in the suburban area were fewer than in other urban gradient areas. In the core urban area, compared with the percentage of increase between two factors, the number of combinations with a 30–50% nonlinear enhancement was dominant. The elements Cu and Sb had fourteen and two interactive combinations with over a 100% linear enhancement, respectively. The element Zn had the largest number (55) of nonlinear increase combinations, which didn’t exceed 50% enhancement. In the suburbs, with the exception of Cd and Sb, the other heavy metal elements had one to four interactive combinations with more than 100% nonlinear enhancement. Furthermore, in the exurbs, three to nine factor combinations with more than 100% nonlinear enhancement were found in the spatial distribution of the metals Cu, Zn, Cd, Sb and Pb. Therefore, we inferred that the nonlinear enhancement interactive effects between two factors were ranked as follows: exurbs > core area > suburbs.

From Table 6, it can be seen that the strong nonlinear factors in the central region that had more than 100% interactive enhancement were mainly concentrated on the distribution of Cu. There was a great interactive enhancement effects among NH_4_^+^/green land area with other topographic and soil factors. The largest interaction of Sb was between altitude and agricultural land area. The large number of strong interactive factors for Cu indicated that the source of influencing factors was complicated.

The number of analytes with more than 100% nonlinear increase interaction in the suburbs was six, which was larger than that in the core area. The strong interaction influencing factors of each element in the Cu-Zn-Ni-Cr group showed similarity, mainly in terms of the interaction between pH and NO_3_^−^-N factors. The interactions between age class and farmland area or land-use category were also large. For the As-Pb group, in addition to the combination of pH and NO_3_^−^-N, there were also strong nonlinear interactions between pH and OM, land use and age of trees.

In the exurbs, the same strong interaction factors of the two elements in the Cr-Ni group were site quality index, humus depth and age of trees. Moreover, the strong interaction factors of the Cu-Zn-Cd-Sb-Pb group also showed this similarity, which was mainly the interaction between topographical factors, topographical and soil factors, soil factors and land use classification, and green land area.

## 4. Discussion

### 4.1. PAC-MLR Methods and Geodetector Model for Source Apportionment of Soil Heavy Metal

The analytes of this study in the environment were mainly derived from natural rocks or sediment. Their contents from this source grows slowly. With the disturbance of human activities, one or more heavy metals in the environment might accelerate accumulating, resulting in much larger difference than the background values in the natural environment, which poses risks to human health and ecosystem elements.

The application of the traditional PCA-MLR method used to analyze heavy metal sources was reported widely [5,6,33,34]. This method classifies pollutants with similar sources into one group and then deduces the possible sources of heavy metals in the group according to the source of the specific pollutants in the emission list. Many studies successfully distinguished between natural and anthropogenic sources [35]. However, the application of PCA-MLR method has been limited by its inability to perform more detailed quantitative analysis of anthropogenic sources. In recent years, the PCA-MLR integrated with GIS spatial analysis method has been gradually developed for the analysis of regional heavy metal sources [5,36,37,38]. The principal factors obtained from PCA are spatially interpolated in order to find possible anthropogenic emission sources and quantify the contribution of different sources. In order to provide better strategies for reducing heavy metal pollution, there is an inevitable demand for improved methods to obtain more detailed impact factor analysis.

According to the theory of the GeoDetector model, if the q value of a certain influencing factor of the spatial distribution of the heavy metal was large, it indicated the factor could determine the distribution of this heavy metal. It represents that the spatial distribution of the factor is similar to the spatial distribution of the heavy metal. Qiao et al. used spatial correlation between the factor and the heavy metal migration quantity in Huangjiang County, China, to prove the reliability of the GeoDetector model for analyzing the impact factor of soil heavy metals [39]. This study used the GeoDetector model to analyze the Cr-Ni group in the central area, the Cr-Ni-Cu-Zn group in the suburbs, and the Cr-Ni group, the Cd-Pb subgroup and the Cu-Zn-Sb subgroup in the exurbs, indicating that the influencing factors were similar. The classification results not only validated the analysis results of the PCA-MLR method, but also provided more detailed information about the human sources. The results also provided assistance for the formulation of the emission reduction strategy. The GeoDetector model also provided possible influencing factors for the single heavy metal group obtained by PCA-MLR method. Moreover, because of the spatial interaction effects of geographic elements, the influence of a single factor on the spatial distribution of heavy metals may not be that important. The nonlinear increase interactions between two factors could be a more powerful determinant [40]. The GeoDetector model provided information about the interaction force of each factor by comparing the sum of the influence of a new feature generated by the superposition of the two factors with the influence of the original factors. This provided comprehensive information for the formulation of subsequent emission reduction strategies [41,42].

The results of this study showed that the interaction combination with strong nonlinear increase in the central region often appeared between the two weak factors, e.g., the topographic factor (q values between 0.001 and 0.002) interacted with the green land area (q = 0.006). The reason for this might be the complex human activities in the core urban area. Strong interactions in the suburbs existed between strong factors and weak factors, e.g., NO_3_^−^N ∩ pH. The explanation for this might be that the dominant human activities in the suburbs were agricultural activities, and the major influencing factors became dominant. In the exurbs, the interaction effect was strong. The strong combinations included the interaction between strong factors and weak factors, e.g., pH∩OM of Cu, and the interaction between two weak factors, e.g., SDi ∩ HD. The reason for this could be fewer human activities and the greater variation of the terrain [43]. Therefore, the strong interaction combinations were mainly between topography and topography, topography and soil, topography and green land characteristic.

### 4.2. Analysis of Pollution Sources

Within a 1 km buffer along the Ningbo Zhangxi River, sources of heavy metals varied with changes in urbanization gradients. The literature reported that the main sources of group Cr-Ni might be traffic pollutants, industry, metal processing, and dust reduction [44,45]. In the center of the study area, the Cr and Ni contents were both low. Therefore, the source of these metals might be natural here. The results of the GeoDetector analysis showed that the distribution of human activity factors was similar to the distribution of Cr and Ni, besides natural factors. In the suburbs, concentrations of the group Cr-Ni were higher than the background values. Their sources were similar to Cu and Zn according to correlation analysis, PCA, and CL analysis methods. The literature considered Zn to be associated with the metal industry and dust and Cu with industrial emissions and municipal wastes [7,46,47]. Therefore, we inferred that the sources of Cr-Ni in suburbs were metal industry emissions. In the exurbs, the source of the Cr-Ni group was similar to As. The low concentrations of these three heavy metals were probable due to the loam soil texture and acidic condition. The red loam developed from the fluvisol containted the lowest level of Cr and Ni than other soil types in Ningbo City [48]. Therefore, the elements Cr, Ni and As may be derived from natural sources.

The concentrations of the Cu-Zn-As-Sb group in the central area were high. The sources of the four metals in this group were similar. It was previously found that As was related to chemical and steel industries and that the chemical properties of Sb were similar to As [2,49]. The content of Sb was infinitesimal in nature with background values of less than 1 mg/kg. In urban area, it was found the waste (household and industry) stream [50] could explain the decreased trend from urban core region to exurbs. However, we found that the concentration of Sb in the central area was more than five times than the background values, which was a large increase. The element Sb and its compounds are widely used in the chemical fields for the production of ceramics, glass, batteries, pyrotechnic materials, printing and dyeing, paints and flame retardants and in pharmaceutical fields [51,52]. Therefore, the pollution sources of the Cu-Zn-As-Sb group could be the automotive metal industry. In the suburbs, the sources of the groups Cu-Zn, As and Sb were different. Except Cu, the average content of other elements was lower than that of the central area. The reason might be that the proportion of industrial sources of As and Sb decreased in the suburbs in contrast to the increase in agricultural sources. In the exurbs, the concentrations of groups Cr-Ni and As were low. However, their sources were different. Since pesticide and phosphate sources contained As, the Cr-Ni group might come from a natural source, while As might come from agricultural source in the exurbs. In the Cu-Zn-Cd-Sb-Pb group, previous studies reported that Cu and Zn were not only related to industrial emissions, but also to agricultural fertilizers [53,54]. The elements Cd and Pb may primarily come from traffic pollutants and accumulate dust [55,56]. In our results, the source of Pb was different from other heavy metals along different urbanization gradients, from which it could be inferred that the source of Pb was always transportation. The source of Cd was different from other metals in the central and suburban areas. According to the records, we speculated that Cd might come from “three wastes”, i.e., emissions from the electroplating, metallurgical industries and transportation in the core urban area [20,57]. It may also relate to metal forging, cadmium-containing sewage from farmland irrigation, and chemical fertilizers in the suburbs. The sources of Cd in the exurbs were agriculture and transportation.

## 5. Conclusions

In this study, soil Cd and Sb increased dramatically compared with the background values of 1990 in Ningbo City. In terms of the impervious surface ratio, the heavy metals had various sources in different urbanization gradients. Multivariate statistics and geostatistics successfully identified similar source metals into four groups in the core urban region: Cr-Ni-Cu, Cu-Zn-As-Sb, Cd and Pb; into five groups in the suburbs: Cr-Ni-Cu-Zn, As, Cd, Sb and Pb; and into three groups in the exurbs: Cr-Ni, Cu-Zn-Cd-Sb-Pb and As. The group Cr-Ni was derived from the metal industry in the suburbs. Cu-Zn-As-Sb came from the automotive metal industry in the core urban area. In the exurbs and the suburbs, the sources of this group were mainly agricultural phosphate fertilizers and pesticides. The element Cd might come from “three wastes”, i.e., emissions from electroplating and metallurgical industries and from transportation in the central area. The element Cd was related to metal forging, cadmium-containing sewage irrigation of farmland, and chemical fertilizers in the suburbs and to agriculture and transportation sources in the exurbs. The main source of Pb in different urbanization gradients was dust accumulation of traffic pollutants. GeoDetector models and spatial analysis not only validated the analysis results of the PCA-MLR method, but also provided more detailed information of artificial sources. For example, in the central urban area, the major influencing factors on the Cr-Ni group not only included natural factors inferred from PCA-MLR results, but also included certain human activity factors. The various major factors of the Cu-Zn-As-Sb group indicated that the sources were similar but different. Furthermore, besides the direct influences, GeoDetector quantified the interactive effects among factors. Interactive combination with strong nonlinear increment changed from between-two-weak factors (topology∩green land area factor) in the central region to between-strong-and-weak factors (NO_3_^−^-N∩pH) in the suburbs. In the exurbs, a stronger interaction effects were observed between strong and weak factors (pH∩organic matter), and between the two weak factors (slope direction∩humus depth). The conclusions of potential sources were made by taking into account the results from PCA-MLR and GeoDetector model, which would be favorable for guiding the formulation of subsequent emission reduction strategies.

The potential toxic elements in urban soil may cause short-term or long-term risks to the health of urban residents through direct or indirect exposure such as ingestion of soil, skin contact and respiratory inhalation. The concentration of Sb in the study area was much higher than the background level. It was likely to have an impact on residents who were exposed to the environment for a long period of time, most notably on lungs, heart organs and residents might be put at a risk of cancer. In addition to industrial products, Sb emission sources were reported to be mainly related to urban wastes. Ningbo City should enhance the monitoring and control of Sb content in industrial and residential waste and the leachate. For Cd, the different contents of urbanization gradients were not significant. According to above analysis results, the city managers should pay attention to the different sources of pollution across the urban gradient, such as the soil pollution caused by sewage irrigation, agricultural non-point source pollution, and the transportation pollution in the exurb besides the agricultural pollution. The pollution mitigation measure could be the plantation of the local plant species which are easy to enrichment of Pb and Cd on the road sides to reduce the pollution of the soil and the risks for the local residents.

## Figures and Tables

**Table 1:** Environmental quality standard for soils(mg/kg) (GB 15168-1995) in China.

Heavy Metal	Class I	Class II			Class III
Cr≤	90	150	200	250	300
Ni≤	40	40	50	60	200
Cu≤	35	50	100	100	400
Zn≤	100	200	250	300	500
As≤	15	20	25	30	30
Cd≤	0.20	0.30	0.30	0.60	1.0
Pb≤	35	250	300	350	500

**Table 2:** Accuracy evaluation of the spatial interpolation.

	Cr	Ni	Cu	Zn	As	Cd	Sb	Pb
Mean Prediction Errors	−2.3668	−0.1954	0.1942	1.0176	0.1171	0.0022	−0.0927	−1.1284
RMSE	72.9	14.8	27.4	14.6	3	0.3	5.5	54.3

**Table 3:** Correlations among heavy metal in three urbanization gradients.

	Factors	pH	OM	NH_4_^+^	NO_3_^−^-N	Cr	Ni	Cu	Zn	As	Cd	Sb	Pb
Core urban region	pH	1.000	0.092	0.313	−0.292	0.025	0.082	0.072	0.115	−0.091	0.321	−0.133	−0.138
	OM		1.000	0.008	0.099	0.285	0.255	0.606 *	0.477	0.442	0.535	0.154	0.265
	NH_4_^+^			1.000	−0.082	0.768 **	0.835 **	0.306	−0.101	0.168	−0.232	−0.075	−0.197
	NO_3_^−^-N				1.000	−0.070	−0.094	−0.208	−0.191	−0.150	−0.184	−0.162	0.008
	Cr					1.000	0.967 **	0.697 **	0.186	0.517	0.127	0.286	0.145
	Ni						1.000	0.651 *	0.203	0.534	0.093	0.230	0.109
	Cu							1.000	0.695 **	0.645 *	0.470	0.637 *	0.370
	Zn								1.000	0.680 *	0.534	0.446	0.487
	As									1.000	0.414	0.499	0.221
	Cd										1.000	0.240	0.184
	Sb											1.000	0.004
	Pb												1.000
Suburb	pH	1.000	−0.145	−0.152	0.364	−0.145	−0.348	−0.393	−0.305	−0.178	−0.420	−0.333	−0.346
	OM		1.000	0.103	0.600	0.340	0.427	0.461	0.479	−0.200	0.052	0.035	0.231
	NH_4_^+^			1.000	0.055	−0.207	−0.094	−0.106	−0.045	−0.568	0.741 *	−0.026	−0.059
	NO_3_^−^-N				1.000	−0.015	0.218	0.108	0.152	−0.276	−0.166	−0.594	0.174
	Cr					1.000	0.746 *	0.735 *	0.731 *	0.158	0.225	0.539	−0.198
	Ni						1.000	0.921 **	0.954 **	0.226	0.287	−0.009	−0.230
	Cu							1.000	0.972 **	0.439	0.116	0.164	−0.012
	Zn								1.000	0.284	0.211	0.071	−0.218
	As									1.000	−0.558	0.110	0.336
	Cd										1.000	0.185	−0.360
	Sb											1.000	0.164
	Pb												1.000
Exurb	pH	1.000	−0.292	−0.664	0.367	0.700	0.738*	0.591	0.618	0.641	0.598	0.711 *	0.617
	OM		1.000	0.220	−0.545	0.073	−0.045	0.459	0.460	−0.471	0.478	0.381	0.341
	NH_4_^+^			1.000	0.160	−0.562	−0.511	−0.717 *	−0.689	−0.831*	−0.405	−0.695	−0.641
	NO_3_^−^-N				1.000	0.356	0.383	−0.323	−0.342	0.051	−0.352	−0.240	−0.426
	Cr					1.000	0.956 **	0.704	0.647	0.565	0.384	0.667	0.350
	Ni						1.000	0.610	0.571	0.651	0.382	0.599	0.301
	Cu							1.000	0.988 **	0.456	0.809 *	0.970 **	0.855 **
	Zn								1.000	0.415	0.869 **	0.990 **	0.908 **
	As									1.000	0.207	0.433	0.340
	Cd										1.000	0.889 **	0.928 **
	Sb											1.000	0.925 **
	Pb												1.000
Whole study area	pH	1	−0.352	0.194	0.207	0.173	0.293	0.297	0.358	0.239	0.331	0.061	0.075
	OM		1	−0.054	−0.017	0.011	−0.028	0.372 *	0.238	−0.002	0.331	0.06	0.094
	NH_4_^+^			1	0.061	0.043	0.326	0.077	−0.02	−0.033	0.044	−0.034	−0.056
	NO_3_^−^-N				1	0.117	0.245	−0.014	−0.05	−0.098	−0.142	−0.117	0.149
	Cr					1	0.773 **	0.504 **	0.132	0.018	0.047	0.017	−0.026
	Ni						1	0.604 **	0.275	0.237	0.115	0.09	0.022
	Cu							1	0.588 **	0.324	0.444 *	0.322	0.228
	Zn								1	0.678 **	0.600 **	0.480 **	0.233
	As									1	0.328	0.492 **	0.205
	Cd										1	0.281	0.052
	Sb											1	0.019
	Pb												1

**Table 4:** Principal component factor scores and eigenvalues of factor loadings in core urban region.

Elements	Core Urban			Whole Region		
	Factor 1	Factor 2	Factor 3	Factor 1	Factor 2	Factor 3
Cr	0.739	−0.638	0.136	0.473	0.800	−0.038
Ni	0.718	−0.656	0.146	0.622	0.688	−0.014
Cu	0.940	−0.002	−0.020	0.827	0.253	0.096
Zn	0.748	0.543	0.057	0.844	−0.335	0.002
As	0.830	0.068	−0.124	0.684	−0.406	0.002
Cd	0.522	0.500	−0.122	0.611	−0.306	−0.183
Sb	0.616	0.117	−0.589	0.561	−0.397	−0.322
Pb	0.393	0.379	0.764	0.255	−0.179	−0.927
Initial Eigenvalues	4.002	1.545	1.005	3.228	1.738	1.009
Variance %	50.021	19.310	12.559	40.347	21.727	12.607
Cumulative %	50.021	69.331	81.890	40.347	62.074	74.680

**Table 5:** The contributions of 17 factors to the concentrations of eight heavy metals.

Urban Gradient	Element	SDi	SPo	SDe	Ele	pH	NH_4_^+^	NO_3_^−^-N	OM	ST	HD	SI	GLAr	DS	Ag	1CLU	2CLU	AAr	Iar	Tar	RRL	NTL
Core urban region	Cr	0.095	0.095	0.101	0.002	0.043	**0.555**	**0.213**	**0.193**	**0.272**	0.115	0.037	0.022	0.015	0.027	0.018	**0.194**	0.065	0.001	0.038	0.024	0.162
	Ni	0.087	0.087	0.094	0.000	0.018	**0.657**	**0.162**	**0.134**	**0.220**	0.100	0.021	0.025	0.013	0.026	0.033	**0.173**	0.084	0.001	0.040	0.009	0.114
	Cu	0.002	0.002	0.001	0.002	**0.094**	**0.078**	0.036	**0.221**	0.033	0.006	0.014	0.006	0.016	0.021	0.015	**0.102**	0.035	0.002	0.018	0.012	**0.263**
	Zn	0.156	0.156	0.151	0.000	**0.435**	**0.378**	0.144	**0.376**	**0.298**	**0.168**	0.040	0.015	0.026	0.023	0.010	0.075	0.006	0.002	0.010	0.002	0.095
	As	0.072	0.072	0.070	0.000	0.079	**0.169**	**0.147**	**0.396**	**0.180**	0.077	0.035	0.011	0.028	0.022	0.084	**0.150**	0.090	0.001	0.005	0.003	0.025
	Cd	0.042	0.042	0.047	0.000	0.030	0.032	**0.124**	**0.088**	**0.056**	**0.057**	0.006	0.004	0.026	0.012	0.013	0.046	0.021	0.001	0.003	0.007	**0.106**
	Sb	**0.156**	**0.156**	0.150	0.000	**0.373**	0.038	0.095	**0.161**	**0.157**	**0.156**	0.006	0.006	0.014	0.014	0.002	0.091	0.001	0.002	0.011	0.005	**0.333**
	Pb	0.013	0.013	0.012	0.001	**0.156**	**0.085**	**0.085**	**0.182**	**0.101**	0.013	0.022	0.004	0.020	0.010	0.002	0.026	0.001	0.004	0.015	0.035	0.015
Suburb	Cr	**0.163**	**0.179**	**0.163**	NA	0.008	**0.383**	**0.164**	0.124	**0.188**	0.162	0.032	0.001	0.026	0.015	0.010	0.107	0.023	0.001	0.003	0.018	0.075
	Ni	0.079	**0.110**	0.079	NA	0.050	**0.445**	**0.150**	**0.190**	**0.117**	0.085	0.035	0.001	0.039	0.019	0.010	0.073	0.004	0.002	0.006	0.015	0.069
	Cu	0.060	**0.113**	0.060	NA	0.052	**0.361**	**0.140**	**0.289**	**0.131**	0.082	0.048	0.002	0.056	0.015	0.008	0.073	0.014	0.000	0.004	0.018	0.017
	Zn	0.058	**0.096**	0.058	NA	0.036	**0.343**	**0.164**	**0.284**	**0.121**	0.079	0.035	0.002	0.052	0.013	0.007	0.062	0.010	0.000	0.008	0.019	0.023
	As	0.004	**0.065**	0.004	NA	0.003	**0.600**	**0.169**	0.041	**0.115**	0.026	0.052	0.000	0.073	0.011	0.000	0.061	0.050	0.001	0.004	0.016	**0.104**
	Cd	0.015	0.029	0.015	NA	0.072	**0.699**	**0.350**	**0.122**	**0.097**	0.018	0.016	0.000	0.065	0.013	0.010	0.054	**0.101**	0.003	0.005	0.022	0.017
	Sb	0.086	0.102	0.086	NA	0.030	0.072	**0.202**	**0.251**	**0.102**	0.088	0.016	0.003	0.030	0.007	0.005	**0.103**	0.026	0.002	0.003	0.004	**0.268**
	Pb	0.015	0.036	0.015	NA	0.022	**0.425**	**0.219**	**0.198**	0.031	0.022	0.016	0.001	0.025	0.009	0.003	0.047	**0.213**	0.000	0.005	0.010	**0.407**
Exurb	Cr	0.043	0.048	0.057	0.191	**0.583**	**0.217**	**0.337**	**0.143**	0.006	0.020	0.042	0.082	0.136	0.055	0.123	**0.200**	0.090	NA	NA	0.046	0.075
	Ni	0.059	0.064	0.076	0.225	**0.519**	**0.416**	**0.420**	0.174	0.004	0.027	0.049	0.106	0.157	0.055	0.156	**0.242**	**0.192**	NA	NA	0.046	0.103
	Cu	0.010	0.063	0.014	0.054	**0.314**	**0.114**	0.036	**0.092**	0.001	0.001	0.004	0.016	0.076	0.055	0.066	**0.111**	0.017	NA	NA	**0.206**	0.008
	Zn	0.010	0.054	0.012	0.054	**0.361**	**0.122**	0.041	0.070	0.001	0.002	0.013	0.012	**0.084**	0.052	0.058	**0.108**	0.025	NA	NA	**0.187**	0.008
	As	0.013	0.085	0.073	0.069	**0.608**	**0.391**	0.025	**0.353**	0.000	0.005	0.031	0.018	0.087	0.033	**0.177**	**0.193**	0.030	NA	NA	0.111	0.003
	Cd	0.023	0.025	0.056	**0.142**	**0.563**	0.077	0.066	**0.111**	0.001	0.002	0.098	**0.030**	0.130	0.044	0.051	**0.135**	0.047	NA	NA	0.097	0.002
	Sb	0.014	0.043	0.018	0.088	**0.513**	**0.134**	0.007	0.055	0.000	0.001	0.049	0.012	**0.113**	0.056	0.064	**0.115**	0.023	NA	NA	**0.170**	0.006
	Pb	0.012	0.033	0.020	0.084	**0.467**	**0.158**	0.055	0.076	0.003	0.004	0.072	0.015	**0.125**	0.050	0.058	**0.124**	0.049	NA	NA	**0.173**	0.001

SDi is the slope direction; SPo is the slope position; SDe is the slope degree; Ele is the elevation; OM is the organic matter; ST is soil texture; HD is the humus depth; SI is the site index; GLAr is the green land area; DS is dominant species; Ag is the age of the dominant species; 1CLU is the first-class land use classification; 2CLU is the second-class land use classification; AAr is the agriculture area; Iar is the industry area; Tar is the transportation area; RRL is the rural residential land; NTL is the nighttime light value. The boldface numbers represent the top five factors.

**Table 6:** Combinations of nonlinear increase more than 100%.

Study Area	Heavy Metal	Interactive Effect Combinations
Core urban	Cu	SDe∩SDi; SDe∩SPo; NH_4_^+^∩SDi; NH_4_^+^∩SPo; NH4∩SDe; NH_4_^+^∩pH; NH_4_^+^∩ST; NH_4_^+^∩HD; NH_4_^+^∩NO_3_^−^-N; GLAr∩SDi; GLAr∩SPo; GLAr∩SDe; GLAr∩NO_3_^−^-N; GLAr∩HD; NO_3_^−^-N∩LU1
	Sb	Ele∩AAr;
Suburb	Cr	pH∩NO_3_^−^-N;
	Ni	pH∩NO_3_^−^-N;
	Cu	pH∩NO_3_^−^-N; AAr∩Ag;
	Zn	pH∩NO_3_^−^-N; LU1∩Ag; AAr∩Ag;
	As	pH∩NO_3_^−^-N; pH∩OM; pH∩LU1; LU1∩IAr;
	Pb	pH∩NO_3_^−^-N; pH∩OM; LU1∩Ag; SD∩Ag;
Exurb	Cr	HD∩SI; SI∩Ag; RRL∩OM;
	Ni	HD∩SI; SI∩Ag;
	Cu	SDi∩SPo; SDi∩SDe; SDi∩HD; SDi∩SD; SDe∩SI; Ele∩OM; pH∩OM; LU1∩OM;
	Zn	SDi∩SPo; SDi∩SDe; SDi∩HD; SDe∩OM; LU1∩OM; LU2∩OM; GLAr∩OM;
	Cd	SDi∩SPo; SDi∩HD; Ag∩SPo;
	Sb	SDi∩SPo; SDi∩SDe; SDi∩HD; SDe∩NO_3_^−^-N; SDe∩OM; Ele∩OM; GLAr∩NO_3_^−^-N; OM∩NO_3_^−^-N; LU1∩NO_3_^−^-N;
	Pb	SDi∩SPo; SDi∩SDe; SDi∩HD; SDe∩OM; Ele∩NO_3_^−^-N; Ele∩OM;

PS: The abbreviations are the same as Table 5.

## Supplementary Materials

The following are available online at http://www.mdpi.com/1660-4601/15/10/2175/s1. Table S1: Source of the samples; Tabel S2: Operational parameters of the analysis by ICP-MS; Table S3: Background values (mg/kg) of eight heavy metals in Ningbo City in 1990; Figure S1: The FMPI soil texture and Food and Agriculture Organization (FAO90) soil type from Harmonized World Soil Database, HWSD in study area; F2: The spatial distributions of pH, organic matter, NH_4_^+^ and NO_3_^-^-N during the urbanization gradients; Figure S3
