# Supplementary material for: Analysis of Heavy Metal Sources in the Soil of Riverbanks Across an Urbanization Gradient
Source: Int J Environ Res Public Health. 2018 Oct 4;15(10):2175. doi: 10.3390/ijerph15102175 (PMC6209923; doi:10.3390/ijerph15102175)
Supplement: Supplementary file 1 [file ijerph-15-02175-s001.pdf]

Table S1. Source of the samples

Urban gradient	Number	Longitude	Latitude
Core urban	ZX1	121.6136111	29.8980556
Core urban	ZX2	121.6016667	29.9086111
Core urban	ZX3	121.5913889	29.8961111
Core urban	ZX4	121.6144444	29.8988889
Core urban	ZX5	121.5736111	29.8894444
Core urban	ZX6	121.5641667	29.8966667
Core urban	ZX7	121.5616667	29.8788889
Core urban	ZX8	121.5533333	29.8747222
Core urban	ZX9	121.5480556	29.8533333
Core urban	ZX10	121.5438889	29.8572222
Core urban	ZX11	121.5347222	29.8477778
Core urban	ZX12	121.5286111	29.8450000
Core urban	ZX13	121.5097222	29.8858333
Suburb	ZX14	121.5225000	29.8058333
Suburb	ZX15	121.5150000	29.8077778
Suburb	ZX16	121.4950000	29.8055556
Suburb	ZX17	121.4822222	29.7736111
Suburb	ZX18	121.4616667	29.7916667
Suburb	ZX19	121.4375000	29.7800000
Suburb	ZX20	121.4086111	29.7752778
Suburb	ZX21	121.3863889	29.7758333
Exurb	ZX22	121.3636111	29.7647222
Exurb	ZX23	121.3452778	29.7775000
Exurb	ZX24	121.3341667	29.7741667
Exurb	ZX25	121.6202778	29.9047222
Exurb	ZX26	121.3161111	29.7969444

Table S1. *Cont.*

Exurb	ZX27	121.3208333	29.7844444
Exurb	ZX28	121.3008333	29.8183333
Exurb	ZX29	121.2919444	29.8166667
Exurb	ZX30	121.2655556	29.8430556

Table S2. Operational parameters of the analysis by ICP-MS

**Parameters**	**Value**
RF power (W)	1550
Nebulizer gas flow (L min^{-1})	1.04
Auxiliary gas flow (L min^{-1})	0.8
Cooling gas flow (L min^{-1})	14
Extraction Lens 2 (V)	–106.7
CCT Focus Lens (V)	–11.40
Sampling cone (mm)	1.0, Ni cone
Skimmer cone (mm)	0.5, Ni cone
Isotopes monitored	52Cr, 60Ni, 63Cu, 66Zn, 75As, 11Cd, 121Sb, 208Pb
Dwell time (ms)	30
Acquisition mode	Peak jumping
Collision gas (He, mL min^{-1})	4.153
Resolution	Normal

Table S3. Background values (mg/kg) of eight heavy metals in Ningbo City in 1990

**Element**	**Number**	**Order statistics**					**Arithmetic mean**	**SD**
		**Minimum**	**0.25**	**Median**	**0.0075**	**Maximum**		
Cr	82	10.800	37.300	63.300	77.100	190.400	62.100	28.470
Ni	81	15.200	23.400	35.400	39.800	50.700	32.600	9.390
Cu	82	3.200	10.300	23.500	28.000	59.900	21.100	10.870
Zn	82	28.500	67.100	82.100	111.000	344.000	99.800	56.980
As	82	1.400	5.000	6.850	9.000	19.100	7.200	3.130
Cd	82	0.010	0.067	0.112	0.156	0.427	0.123	0.070
Sb	17	0.610	0.780	0.910	1.030	1.480	0.920	0.203
Pb	82	12.900	21.750	27.000	31.750	80.200	28.100	10.140

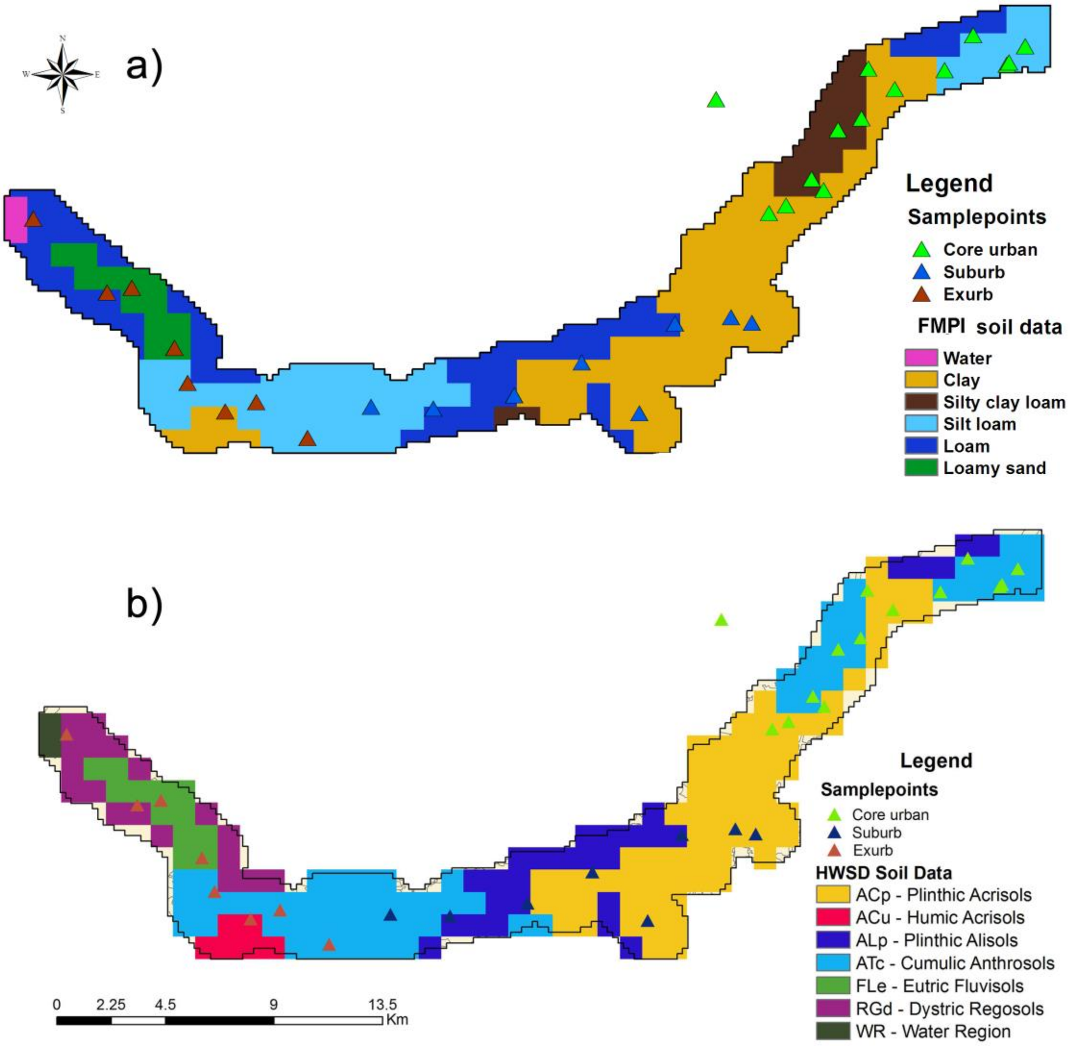

Figure S1. The FMPI soil texture and Food and Agriculture Organization (FAO90) soil type from Harmonized World Soil Database, HWSD in study area. HWSD Sources: Fischer, G.;, F. Nachtergaele, S. Prieler, H.T. van Velthuizen, L. Verelst, D. Wiberg, check names 2008. Global Agro-ecological Zones Assessment for Agriculture (GAEZ 2008). IIASA, Laxenburg, Austria and FAO, Rome, Italy.

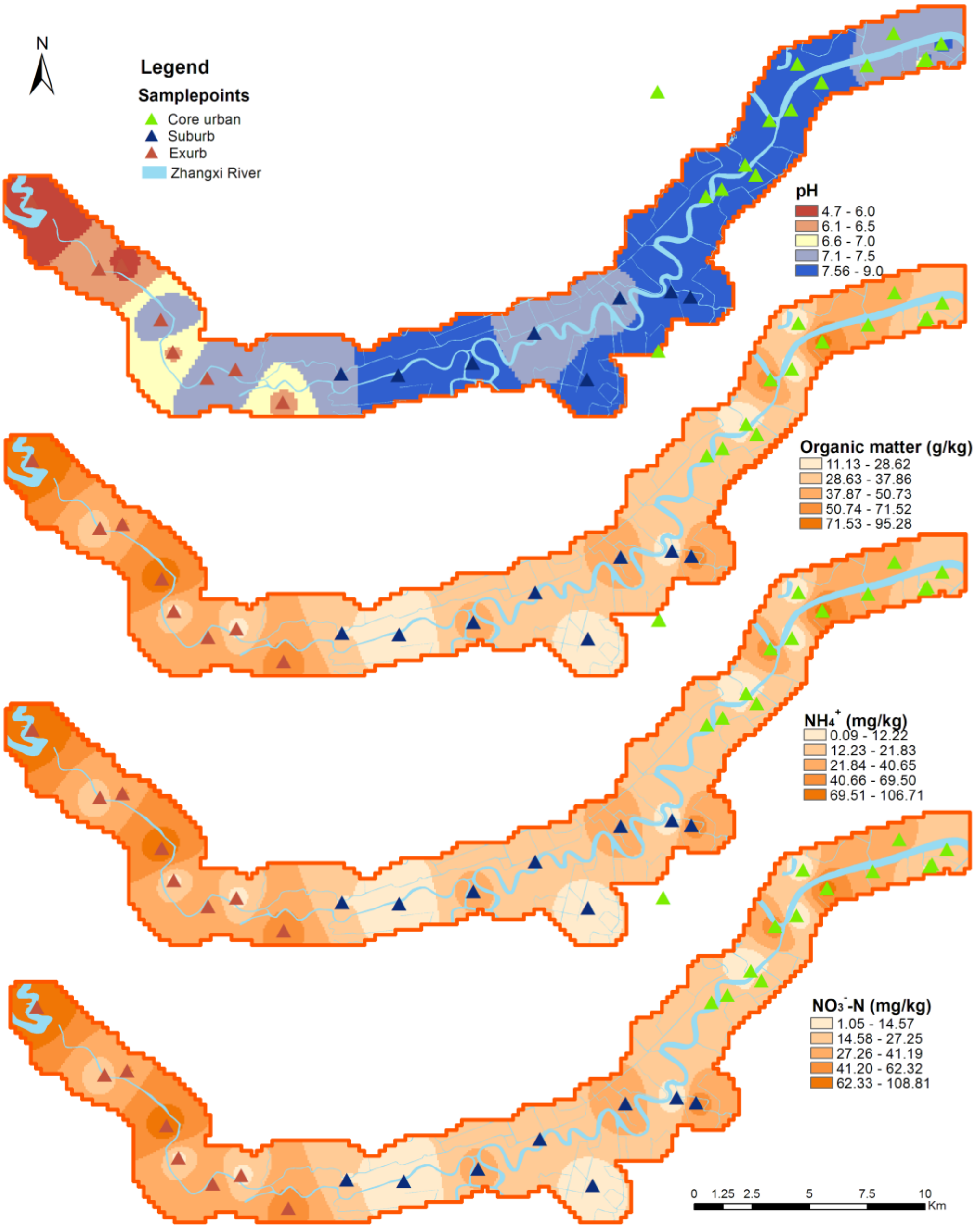

Figure S2. The spatial distributions of pH, organic matter, NH_4^+ and NO_3^--N during the urbanization gradients

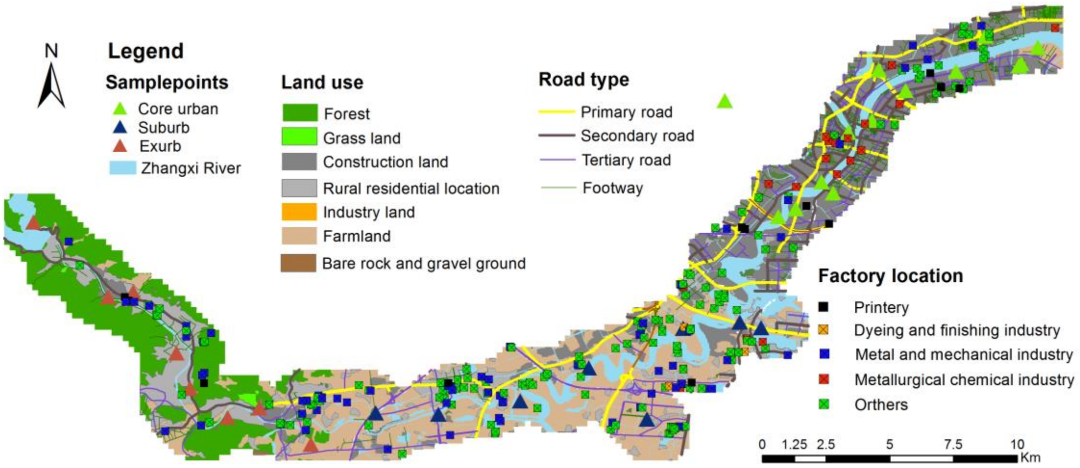

Figure S3. Land use, road network and factory locations in the study area
